# Making a Home for Individuals With Serious Mental Illness: A Systematic Review

**DOI:** 10.1177/00207640251387785

**Published:** 2025-11-04

**Authors:** Emi Patmisari, Yunong Huang, Ros Wong, Mark Orr, Sumathi Govindasamy, Emily Hielscher, Helen McLaren

**Affiliations:** 1 College of Business, Law and Government, Flinders University, Adelaide, SA, Australia; 2 College of Education, Psychology and Social Work, Flinders University, Adelaide, SA, Australia; 3 Flourish Australia, Sydney, NSW, USA; 4 QIMR Berghofer Medical Research Institute, Herston, QLD, Australia; 5 Faculty of Medicine, School of Public Health, The University of Queensland, Brisbane, Australia; 6 School of Allied Health, Australian Catholic University, Fitzroy, VIC, Australia

**Keywords:** SMI, severe mental illness, assisted independent housing, assisted independent living, residential care facility

## Abstract

**Background:**

Housing is widely recognised as a key social determinant of mental health and recovery, yet the concept of *home* remains under-theorised and inconsistently applied in practice and policy.

**Aims:**

The aim of this systematic review is to examine *home* for people with serious mental illness (SMI), focussing on various stakeholder perspectives reported in literature.

**Methods:**

Records from CINAHL, Emcare, ProQuest, PsycINFO, PubMed, Scopus, and Web of Science (*n* = 5,309) were double screened, resulting in 26 peer-reviewed studies for inclusion. Thematic analysis identified fifteen themes synthesised according to perspectives of four different populations: individuals with SMI; their family members; landlords; staff; and sector experts.

**Results:**

Contrary to a house, home was described as shaped by emotional safety, personal control, daily routines, and the quality of social relationships. Findings underscored the need for relational, flexible, and context-sensitive housing approaches that support autonomy, continuity, and belonging.

**Conclusions:**

This review contributes to a more nuanced understanding of home as a multi-dimensional and negotiated concept, from the perspective of multiple stakeholders, with implications for mental health policy, service design, and future research.

## Introduction

Supported housing for people with SMI refers to self-contained units on one site with on-site staff present during set hours to provide social, emotional, and practical support; assisted independent housing refers to community-based, self-contained accommodation with flexible outreach support; and residential care facility refers to accommodation with 24-hr on-site supervision, often in shared or institutional settings. Safe, stable, and supportive housing is essential for mental health recovery. However, people with serious mental illness (SMI) commonly face housing instability which worsens psychiatric symptoms, increases hospitalisations, and reduces engagement with support services. When unable to secure public housing, many experience private rental market discrimination (Culhane et al., 2002; Patterson, 2020). Research shows that housing insecurity leads to distress, disengagement, and acute psychiatric episodes (Leopold, 2019). Understanding intersections between housing and psychiatric wellbeing is crucial in designing effective, recovery-oriented interventions.

Support to people with SMI into housing extends well beyond the immediate physical living space (Newman & Goldman, 2008, 2009). Appropriate housing requires stability, it should foster personal autonomy, social inclusion, and meaningful activity (Doroud et al., 2018). Based on recovery-oriented approaches, housing is more than a shelter. It is a home that enables individuals to reclaim their identities, engage in meaningful activities, and build supportive relationships (Davidson, 2016; Padgett, 2007; Rowe & Davidson, 2016). A home enables security and sense of belonging in one’s community. Both are essential for longer-term recovery, as highlighted by the concept of ontological security indicating that stable and predictable living conditions reduce psychological distress and promote wellbeing (Atkinson & Jacobs, 2017; Dupuis & Thorns, 1998; Shaw, 2004).

The socio-ecological model (Bronfenbrenner, 2000) illustrates how personal relationships and broader policy structures interact to shape housing, stability, and recovery, influencing both lived experience and mental health outcomes. Collaborative stakeholder engagement is essential when designing housing systems for individuals with serious mental illness (SMI), as system-level barriers demand community-driven solutions beyond traditional, locality-based approaches (Sylvestre et al., 2006). Some service models reflect this shift; for instance, Housing First challenges conventional notions of ‘housing readiness’ by positioning stable housing as a prerequisite for recovery and community inclusion (Tsemberis et al., 2004). These approaches underscore that housing is foundational to recovery, not a reward for it.

However, research on housing interventions for people with SMI remains narrowly focused on clinical outcomes, often prioritising symptom reduction over broader psychosocial recovery (Kyle & Dunn, 2008; McPherson et al., 2018). The literature is dominated by accommodation type classifications (Friesinger et al., 2019; Jose et al., 2021), rather than examining housing factors that foster a sense of home. While Ketola et al. (2022) found assisted independent housing improved psychiatric stability, others reported no significant differences in quality of life or social outcomes across settings (Brunt & Hansson, 2004; Dehn et al., 2022). These discrepancies expose key gaps, as most studies overlook the subjective experiences central to recovery. Richter and Hoffmann (2017) called for evaluations that consider psychiatric stabilisation alongside service-user preferences, community engagement, and personal recovery. There is limited qualitative inquiry in this area, underscoring the need for a more holistic, person-centred understanding of housing and mental health.

Service-user perspectives consistently advocate for comprehensive, recovery-oriented approaches that recognise home as vital to psychiatric wellbeing, social inclusion, and self-agency (e.g. Friesinger et al., 2019; Jose et al., 2021; Krotofil et al., 2018; Richter & Hoffmann, 2017). Incorporating diverse stakeholder voices, including individuals with SMI, their families, property stakeholders, and service providers, is essential for developing sustainable, high-quality housing solutions. This systematic review addresses key gaps by documenting factors that help transform assisted independent or community-based residential accommodation into a home. Our mixed-methods synthesis captures the experiences of service users, providers, and the wider community, mapping transformative elements that support recovery and enable citizenship.

## Methods

This systematic review followed the PRISMA guidelines and checklist (Page et al., 2021), protocol registration with PROSPERO (CRD42013434987), and review question: ‘What makes a living space into a *home* for people with SMI?’

### Search Strategy

Search terms were formulated using the PICO framework (van Loveren & Aartman, 2007). Seven electronic journal databases were systematically searched on 31 December 2024 for relevant studies: CINAHL, Emcare, ProQuest, PsycINFO, PubMed, Scopus, and Web of Science. Search terms, truncation. Boolean operators were tailored to specific requirements of each database (see Supplemental Table S1). One author undertook searches (EP), checked by co-authors (HM, RW), with forward/backward searching (EP, HM, RW) searching of studies marked for inclusion via Google Scholar on 15 January 2025.

### Selection Criteria

Studies in English language of people with SMI (generally or specific disorders, e.g. schizophrenia, bipolar disorder, major depression, and PTSD) were included. As were quantitative, qualitative, and mixed methods studies examining any type of intervention or strategy with focus on home. There were no restrictions to housing type (e.g. room, apartment, or house; single or shared occupancy) or context (e.g. owner-occupier, private rental, or specialised community housing). Adults and youth populations were included (we accepted definitional variations of youth). Studies on obtaining housing, custodial settings, or long-term hospitalisation facilities, were excluded. No publication year or country restrictions were applied.

### Screening and Study Selection

**Figure 1.**
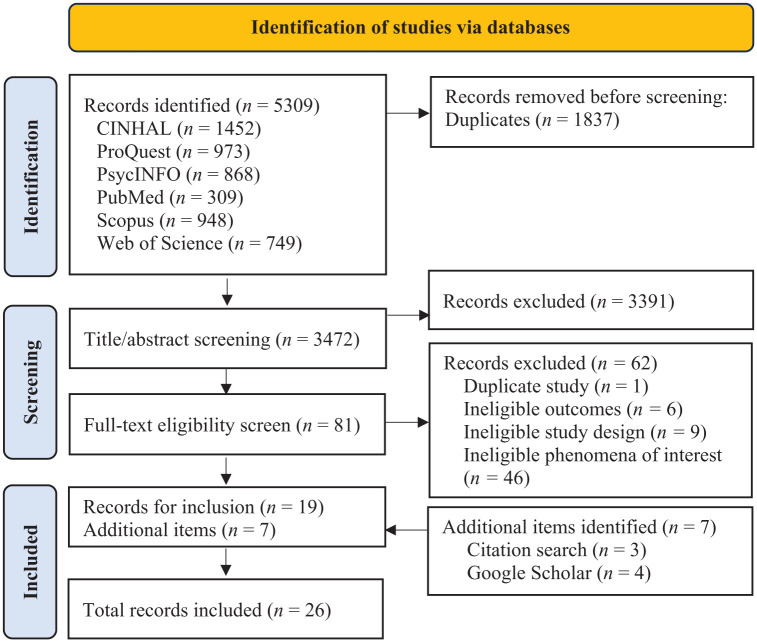
PRISMA flow diagram of studies included.

Search results totalling 4,882 records were exported to EndNote (v.21) automating 1,837 duplicate removals. The remaining 3,472 were exported to JBI SUMARI (Munn et al., 2019) for screening. Title and abstract screening (HM and RW) and conflict resolutions (EP) facilitated 3391 exclusions, leaving 81 records for full-text double screening (EP, HM, RW, and YH). Sixty-two conflicts were removed (EP and HM). Twenty-three studies, and three from forward/backward searching, totalled 26 studies for inclusion (Figure 1).

### Quality Appraisal

Each record was independently appraised by two authors (EP and RW) using the Mixed Methods Appraisal Tool (MMAT), and conflict resolve by one author (HM). Since all studies were empirical, Part 1 of MMAT screening was not required (Hong et al., 2018). The MMAT assesses methodological quality of qualitative, randomised controlled trials, non-randomised, quantitative descriptive, and mixed methods studies. Ratings were assigned to each study and scored: ‘yes’ scores 1, and ‘no’ or ‘can’t tell’ scores 0 (Pluye et al., 2009). Scores were added, converted to percentages, and categorised as low (under 35%), medium (36%–70%), or high (71%–100%) methodological quality (Table 1). Appraisal was used to highlighted limitations in the body of knowledge, not to exclude poor quality studies.

**Table 1.** Mixed Methods Appraisal Tool (MMAT).

Qualitative studies	Study design	Is the qualitative approach appropriate to answer the research question?	Are the qualitative data collection methods adequate to address the research question?	Are the findings adequately derived from the data?	Is the interpretation of results sufficiently substantiated by data?	Is there coherence between qualitative data sources, collection, analysis, and interpretation?	Score
Bengtsson-Tops et al. (2014)	Phenomenology	Y	Y	Y	Y	Y	5/5
Forchuk et al. (2023)	Ethnography	Y	Y	Y	Y	Y	5/5
Fossey et al. (2020)	Narrative	Y	Y	Y	Y	Y	5/5
Hill et al. (2010)	Grounded theory	Y	Y	Y	Y	Y	5/5
Lindström et al. (2011)	Phenomenology	Y	Y	Y	Y	Y	5/5
Lindström et al. (2013)	Narrative	Y	Y	Y	Y	Y	5/5
Molin et al. (2023)	Qualitative descriptive	Y	Y	Y	Y	Y	5/5
Piat and Seida (2018)	Qualitative descriptive	Y	Y	Y	Y	Y	5/5
Piat et al. (2020)	Qualitative descriptive	Y	Y	Y	Y	Y	5/5
Piat et al. (2017)	Qualitative descriptive	Y	Y	Y	Y	Y	5/5
Rönngren et al. (2018)	Qualitative descriptive	Y	Y	Y	Y	Y	5/5
Steno and Jønsson (2023)	Ethnography	Y	Y	Y	Y	Y	5/5
van Genk et al. (2024)	Qualitative descriptive	Y	Y	Y	Y	Y	5/5
Quantitative studies	Quantitative descriptive	Is the sampling strategy relevant to address the research question?	Is the sample representative of the target population?	Are the measurements appropriate?	Is the risk of nonresponse bias low?	Is the statistical analysis appropriate to answer the research question?	Score
Brunt and Rask (2018)	Descriptive cross-sectional	Y	Y	Y	CT	Y	4/5
De Heer-Wunderink et al. (2012)	Correlational cross-sectional	Y	Y	Y	CT	Y	4/5
Marcheschi et al. (2013)	Cross-sectional analytic	Y	Y	Y	CT	Y	4/5
Marcheschi et al. (2014)	Cross-sectional analytic	Y	Y	Y	CT	Y	4/5
Marcheschi et al. (2015)	Cross-sectional analytic	Y	Y	Y	CT	Y	4/5
	Quantitative non-randomised controlled trial	Are the participants representative of the target population?	Are measurements appropriate regarding both the outcome and intervention (or exposure)?	Are there complete outcome data?	Are the confounders accounted for in the design and analysis?	During the study period, is the intervention administered (or exposure occurred) as intended?	Score
Eklund and Tjörnstrand (2022)	Quasi experimental – longitudinal	Y	Y	Y	N	Y	4/5
	Quantitative randomised controlled trial	Is randomisation appropriately performed?	Are the groups comparable at baseline?	Are there complete outcome data?	Are outcome assessors blinded to the intervention provided?	Did the participants adhere to the assigned intervention?	Score
Gyllensten and Forsberg, (2017)	Cluster-randomised trial	Y	Y	Y	N	Y	4/5
Mötteli et al. (2022)	Pragmatic randomised trial	Y	Y	Y	Y	Y	5/5
Mixed methods studies	Type of study	Is there an adequate rationale for using a mixed methods design to address the research question?	Are the different components of the study effectively integrated to answer the research question?	Are the outputs of the integration of qualitative and quantitative components adequately interpreted?	Are divergences and inconsistencies between quantitative and qualitative results adequately addressed?	Do the different components of the study adhere to the quality criteria of each tradition of the methods involved?	Score
Adamus et al. (2023)	Convergent design	Y	Y	Y	CT	CT	3/5
Felx et al., (2020)	Sequential exploratory design	Y	Y	Y	CT	CT	3/5
Marcheschi et al. (2016)	Convergent design	Y	Y	Y	CT	Y	4/5
Tjörnstrand et al. (2020)	Convergent design	Y	Y	Y	CT	Y	4/5
van Genk et al. (2023)	Convergent design	CT	Y	Y	CT	CT	2/5

### Data Extraction and Preliminary Synthesis

Data extracted from each study included author, year, country, research design, and housing/home-related interventions. Independent thematic analysis (EP and HM) followed Braun and Clarke’s (2022) approach: iterative reading, coding, theme development, and refinement. Due to substantial heterogeneity and inconsistent themes, mainly qualitative data informed the review. Through author deliberation (EP, HM, and YH), themes were organised around four participant perspectives: people with SMI; on-site and visiting staff; family members and landlords; and academic/industry experts (including user panel members). This framework supported the presentation of diverse viewpoints on housing/home contexts, interventions, and experiences.

## Results

Thirteen qualitative, seven quantitative, and six mixed methods studies were included in the review (Table 2). MMAT appraisals showed high quality for qualitative (100%) and quantitative studies (83%), and moderate quality for mixed methods (67%), with overall average of 82%. Most were conducted in Sweden (50%), then Canada (19%), the Netherlands (12%), Australia and Switzerland (8% each), and Denmark (4%). Studies represented at least 1,143 people with SMI, 699 staff, 75 family members and landlords, and 64 academic or industry experts (unclear reporting precluded exact numbers). Nine studies showed 43% women, 56% men, and <1% non-binary (not provided in other studies). All studies focused on adults, with only one study on young adults aged 18 to 28 years (Steno & Jønsson, 2023).

**Table 2.** Summary of Included Studies.

Authors, year, country	Aim	Design/methods	Housing/home interventions	Findings
Adamus et al. (2023) – Switzerland	To examine perspectives of mental health professionals in Assisted Independent Housing and Residential Care Facilities.	Mixed methods, convergent. Online survey of 112 mental healthcare workers (70 women, 41 men, and 1 gender ‘diverse’), mean age 40.3 years, 75.9% had completed higher education.	Assisted Independent Housing (group homes) – and Residential Care Facilities in Switzerland.	Effective supported housing for SMI hinges on stable mental health, user capabilities and motivation, treatment adherence, accessible support, mutual goals, and professional education’s role in care quality. Challenges include affordability, financial limitations, and regulatory barriers.
Bengtsson-Tops et al. (2014) Sweden	To explore the experiences of landlords who have tenants with SMI who lived independently in housing settings.	Qualitative, phenomenology. Open in-depth interviews and thematic latent content analysis, 16 landlords (7 women and 9 men), aged 25–62 years.	Private and public housing settings in urban and rural areas of Sweden. People with SMI live independently in these housing settings without on-site services.	Landlords faced challenges, including property damage, and sanitation issues; complaints about disturbances; safety and management difficulties due to threatening or erratic behaviours; a lack of support from psychiatric and social services in crises; and the need to extend help beyond professional duties, such as conflict mediation, daily task assistance, and activity support.
Brunt & Rask (2018) Sweden	To assess how residents and staff of supported housing facilities perceive the frequency and importance of verbal and social interactions.	Quantitative, descriptive cross sectional. Verbal and Social Interaction Supported Housing questionnaire, 111 supported housing facility tenants with SMI (48 women and 63 men), aged 20 to 82 years, and 223 staff members (145 women and 68 men), aged 20 to 62 years.	Specialised residential care facilities (group homes) designed to provide accommodation and support for people with SMI in Sweden. These facilities aim to offer a more integrated and community-oriented approach to care.	Staff valued interaction frequency and importance more than residents, emphasising relationship building and social activities. Residents focused on trust, social engagement, and autonomy, while staff prioritised a safe, supportive environment, practical skills, and emotional-social well-being.
De Heer-Wunderink et al. (2012) the Netherlands	To investigate the levels of social inclusion among individuals with SMI comparing two different types of psychiatric community housing programs.	Quantitative, correlational cross-sectional. Survey via a service user diary, 255 people with SMI in community housing programs (115 women, 140 men), mean age 43.8 years, and 75 workers.	Comparison of residential care facilities that included daily to 24-hr supervision, with assisted independent housing offering psychosocial support with two weekly visits.	Service users in assisted independent housing engaged in more activities and received more visits than those in residential care facilities, with no difference in vocational participation. Independently living users reported more unmet social needs, yet a majority received support from partners, family, or friends.
Eklund & Tjörnstrand (2022) – Sweden	To evaluate how residents and staff in supported housing perceived an intervention designed to enhance meaningful activities and personal recovery.	Quantitative, non-RCT. Three measurement points, pre- and post-program, and 6–9 months post-completion, 29 people with SMI in supported housing and 43 staff members.	Active in My Home was led by an occupational therapist and comprised five individual and three group sessions.	Both residents and staff were satisfied with the Active in My Home intervention, with staff more positive about its effectiveness; however, it didn’t notably affect residents’ housing satisfaction or their views on recovery support.
Felx et al. (2020) Canada	To develop a comprehensive conceptual model of housing and community-based residential care facilities for people with SMI.	Mixed methods, sequential exploratory. Multidimensional scaling and hierarchical cluster analysis, with at least 49 people with SMI, 43 family members, 46 professionals and industry experts, and 83 staff. Authors estimated ~500 unique participants but overlap across six study phases not provided	Housing and community-based residential care facilities for people with SMI.	Created conceptual models. The final model features 12 clusters: Balanced housing, Quality management, External environment, Tailored services, Linkage interventions, Equality and availability, Organisational qualities, Skills interventions, Daily living support, Personal privacy, Interior environment, and Respectful atmosphere.
Forchuk et al. (2023) Canada	To evaluate tenant experiences and impact on personal growth, development, and community integration during transitions from Homes for Special Care to Community Homes for Opportunity.	Qualitative, ethnography. Focus groups at pre-implementation, transition, and final stages of the Community Homes for Opportunity implementation, 188 people with SMI from 28 group homes.	The intervention involved modernising the existing Homes for Special Care into Community Homes for Opportunity in Southwestern Ontario, Canada.	The shift to Community Homes for Opportunity was positive, enhancing tenants’ quality of life, independence, and empowerment, with improved support, social interactions, and community integration. Challenges included limited space, financial management concerns, and a need for greater program understanding.
Fossey et al. (2020) Australia	To explore the everyday lives and community participation of people with SMI focusing specifically on their housing and neighbourhood experiences.	Qualitative, narrative. 1–3 interviews or focus group per participant, narrative and thematic analysis, 39 people with SMI (18 women and 21 men) living in metropolitan areas receiving community mental health care.	Residing in various housing types, including rental accommodation (some in public housing) and family homes in metropolitan Melbourne, Australia. Participants received outreach support, characterised as individual accommodation without on-site staff, with low/moderate support.	Participants valued housing and neighbourhood qualities, identifying a place as home, interactions, safety, accessibility, and support in defining home. Unsatisfactory housing and low income harmed well-being, with pets and external social activities as coping strategies.
Gyllensten & Forsberg (2017) Sweden	To evaluate the effectiveness of Exergames to increase physical activity habits in communal supported house settings for individuals with SMI.	Mixed methods, sequential explanatory. Cluster-randomised trial study, 73 people with SMI from 18 communal supported houses (43 intervention group and 30 control group), focus groups and interviews with trial participants, 11 staff members, and 1 technical assistant.	The intervention involved Exergames controlled by body movements, contrasting with the control group that used ordinary TV games controlled by hand. The settings were communal supported houses in the social psychiatry system in northern Sweden.	The study showed no notable differences in physical activity or health outcomes between groups post-intervention, with only 5% regularly using Exergames due to technological issues and staff scepticism. Despite enjoyment by some, the intervention’s effectiveness in boosting activity and health was unproven.
Hill et al. (2010) Australia	To explore the process of transitioning to independent housing for individuals with schizophrenia.	Qualitative, grounded theory. Semi-structured interviews and email communications with seven people with SMI (two women and five men) living independently.	The focus was on the transition to independent housing environments in New South Wales, Australia.	The transition to independent housing for individuals with schizophrenia involves three crucial non-linear processes: gaining control, linking illness and place, and developing belonging.
Lindström et al. (2011) Sweden	To explore how people with SMI experience personal changes within a residential care facility.	Qualitative, phenomenology. In-depth interviews with supported housing residents, employing constant comparative analysis for data evaluation, six people with SMI (two women and four men).	The intervention setting was a supported housing residence where each participant lived in a self-contained apartment in northern Sweden.	Supported housing led to ‘occupational transformations’, enhancing residents’ daily engagement, mental illness management, social skills, and motivation through occupational successes.
Lindström et al. (2013) Sweden	To understand how people with SMI interpreted a model for integrated occupational therapy in sheltered or supported housing facilities.	Qualtiative, narrative. Interviews and field observations, narrative analysis to understand the meaning-making processes of 18 people with SMI (7 women and 9 men).	Everyday Life Rehabilitation was implemented in sheltered and supported housing facilities.	‘Rediscovering agency’ involved occupational and identity shifts, highlighting hope, goal attainment, community reintegration, and crucial healthcare support.
Marcheschi et al. (2013) Sweden	To explore how the perceived physical environmental quality of housing facilities for people with SMI influences the perceived social climate.	Quantitative, cross-sectional analytic. Multi-dimensional data collection, 189 participants, comprising 72 people with SMI (40% women and 60% men) and 117 staff members (70% women and 30% men) of Supported-Housing Facilities, and five environmental psychologists.	The study investigated 20 Supported-Housing Facilities located in southern Sweden to ensure variation in terms of home-likeness and localisation (city centre vs. suburb).	Residents and staff in Supported-Housing viewed their social relationships positively, linking physical environment quality to social climate perception. Yet, expert assessments of the environment diverged from these perceptions, with staff more positive about the social climate than residents.
Marcheschi et al. (2014) Sweden	To explore how differences in the quality of the physical environment at various Supported-Housing Facilities (SHFs) influence the perception of supportive qualities.	Quantitative, cross-sectional analytic. Using instruments such as post-occupancy evaluation, and semantic environmental description, 72 people with SMI (40% women and 60% men), 117 staff members (70% women and 30% men), 3 user-group panel members, and 5 environmental psychologists.	Supported-Housing Facilities in Sweden, specifically designed for people with SMI. Facilities were categorised into High-Quality and Low-Quality Supported-Housing Facilities based on physical-environment characteristics.	Higher-quality Supported-Housing Facilities were seen as more supportive and homelike, promoting positive psychosocial processes, with experts and users sharing similar views. High-quality facilities were suburban with better independence options, while lower-quality ones, provided easier community service access. Commonly, SHFs displayed institutional rather than homelike indoor environments, regardless of quality.
Marcheschi et al. (2016) Sweden	To explore the role of architectural characteristics in Supported Housing Facilities in fostering social interactions.	Mixed methods, convergent. Observational using behavioural mapping and event-sampling methods to capture and analyse interactions, 29 people with SMI (14 women and 15 men) and 27 staff (24 women and 3 men).	Supported Housing Facilities with varying levels of perceived physical quality, defined as High Quality and Low Quality.	High-Quality facilities featured dining areas that enhanced social interactions among SHF users, with outdoor access and proximity further boosting social behaviour, unlike corridor areas where no differences were observed.
Marcheschi et al. (2015) Sweden	To investigate how the perceived physical and social-environment qualities of supported housing facilities affect the quality of life of people with SMI.	Mixed methods, convergent. Observational study using a user-centred approach. Instruments used to assess quality of physical and social environments, place attachment, and the quality of life, 72 people with SMI (40% women and 60% men).	20 different housing facilities located in the Scania region of southern Sweden. These were private apartments in a single building with professional workers providing daily, round-the-clock assistance, and support.	Perceived physical and social-environment qualities, along with place attachment, significantly influenced the quality of life for individuals with SMI, with place attachment fully mediating the impact of physical and partially mediating social-environment qualities on life quality.
Molin et al. (2023) – Sweden	To elucidate nurses’ experiences in providing health counselling to people living with SMI in supported housing.	Qualitative, descriptive. Semi-structured individual interviews, inductive content analysis, 8 registered nurses (7 women and 1 men) aged 40–60 years, working in supported housing.	Supported housing for people with SMI in a municipality in northern Sweden.	Nurses were disheartened, facing obstacles from residents’ self-determination rights that limited health counselling effectiveness, struggled to control situations, and saw residents making poor health choices. Lack of caregiver collaboration and feelings of failure in fostering healthier lifestyles were key concerns.
Mötteli et al. (2022) – Switzerland	To evaluate the effectiveness of Assisted Independent Housing (AIH) for non-homeless individuals with SMI.	Quantitative, pragmatic randomised trial. Data collection at baseline, 6- and 12 months, 58 people with SMI (30 AIH service intervention group and 28 control group) receiving regular housing and support services.	The AIH service in Zurich was the intervention, designed as a low-threshold community-based outreach housing rehabilitation service for adults with SMI and related housing problems	After 12 months, the intervention group showed significant living independence and reduced inpatient treatment needs. The AIH service matched regular services in social inclusion and outcomes at a cost of 115 Swiss Francs per participant per month, proving effective in housing rehabilitation and preferred by users, aligning with the UN Disability Rights Convention.
Piat et al. (2017) Canada	To explore the effects of transitioning from supervised to supported housing on SMI recovery and community connections.	Qualitative, descriptive. Data collection by photo-elicitation, 24 people with SMI, aged 18–64 years, who had moved from custodial to supported housing.	Supported housing provided individual apartments with amenities, emphasising consumer control, privacy, and access to resources.	Participants in supported housing reported enhanced quality of life and mental health recovery, highlighting the transition to a sense of home, amenities appreciation, better accessibility, identity shifts, and the significance of therapeutic landscapes in recovery, underscoring the physical, social, and symbolic environment’s role in mental health support.
Piat & Seida (2018) Canada	To explore families’ perspectives on the role of supported housing in the personal recovery of people with SMI.	Qualtiative, descriptive. Interviews about the housing situation of people with SMI, recovery orientation of services, choice and autonomy, and community integration, 16 family members of people with SMI.	The intervention was supported housing across five sites in four Canadian provinces. The facilities offered full tenancy rights and mix of independent housing options, including in apartment buildings with staff support or scattered site units in regular apartments.	Families acknowledged supported housing for aiding tenants in identity reshaping, social reconnections, and life control, but were sceptical about its role in ensuring future employment or complete recovery.
Piat et al. (2020) Canada	To understand how personal choice facilitates the recovery process of people with SMI in supported housing environments.	Qualitative, descriptive. Interviews and thematic analysis, 24 people with SMI, aged 18–64 years.	The intervention is the supported housing model from five supported housing projects across four Canadian cities.	In supported housing, tenant wellbeing and mental health recovery were linked to choices in personal responsibility, social organisation, and home-like autonomy, spanning daily tasks to broader financial and healthcare decisions, crucial for their recovery.
Rönngren et al. (2018) Sweden	To evaluate the acceptability of the lifestyle program for people with SMI receiving housing support.	Qualitative, descriptive. Focus groups interviews, analysed using manifest content analysis, 13 participants with SMI, aged 18 to 65 years, and 6 staff members.	The PHYS/CAT intervention was conducted in a Swedish municipality. An integrated relational, educational, and support component into daily nursing care.	The program was positively received, with 9 out of 13 users completing it, showing benefits in physical activity, social interactions, and lifestyle motivation. Challenges were noted in needing more personalised support, education, maintaining activity, and food logs.
Steno & Jønsson (2023) Denmark	To explore the interactions and relationships of young people with SMI within recovery-oriented social housing facilities.	Qualitative, ethnography. Fieldwork, across 3 years, involving 1 to 3 interviews with each participant, 10 people with SMI (4 women, 6 men, and 1 nonbinary), aged 18–28 years.	Recovery-oriented social housing facilities for young adults with SMI in Denmark.	Residents’ meetings, meant to foster participation in recovery, often made users feel awkward and reluctant, due to feeling alienated. Recognising the diverse ways users engage or disengage is key to understanding recovery in psychosocial rehabilitation.
Tjörnstrand et al. (2020) Sweden	To understand how people with SMI living in supported housing experience their day-to-day lives.	Mixed methods, convergent. Diary of activities, activity frequency calculations and manifest content analysis, 155 people with SMI in supported housing (133 completed diaries), 44% women, 56% men, and 99% single, mean age 48 years.	The SH in this study are congregate residential facilities in Sweden. The accommodation varied from fully-fitted apartments to shared facilities with common areas for group activities. The staff provided support, including counselling, treatment, and daily assistance.	Residents displayed low activity levels, like music and resting. Motivation to leave stemmed from errands and group events. Key experiences included voluntary and compulsory socialisation, overcoming loneliness, activity spurred by environmental and emotional shifts, and varying degrees of support, friendship, and security needs fulfilment.
van Genk et al. (2023) the Netherlands	To identify professional perspectives on essential requirements of Intensive Home Support to people with SMI.	Mixed methods, convergent. Purposive sampling, statistical analysis, and concept mapping, 17 experts (9 women and 8 men) participated in brainstorming, sorting and rating the needs of 14 people with SMI (7 women and 7 men).	Intensive Home Support for people with SMI is an alternative form of outpatient support intended for individuals living independently in the community but requiring intensive support.	The study identified 10 key aspects of Intensive Home Support: housing rights, informal collaboration, community reciprocity, normalisation, citizenship, recovery, sustainable funding, 24/7 flexible support, public and positive health, and integrated home support cooperation.
van Genk et al. (2024) the Netherlands	To explore clients’ experiences of Intensive Home Support (IHS) and identify mechanisms supporting recovery.	Longitudinal qualitative descriptive study. Semi-structured interviews with 42 participants (27 males and 15 females) using a qualitative thematic analysis framed by the CAIMeR theory.	Intensive Home Support (HIS) where services are provided where the person lives, rather than in clinical or institutional settings.	Five critical ingredients of IHS: Working alliance (trust, open communication, continuity, and feeling equal to support workers); Autonomy (empowerment, decision-making, and opportunities for participation); Relationships (with family, friends, and peers); Mental and physical health (continued need for coordinated care); and Housing and environment (independent, quiet, and suitable housing). Trust, recognition, and security were tightly linked to the qualities of the living environment.

**Figure 2.**
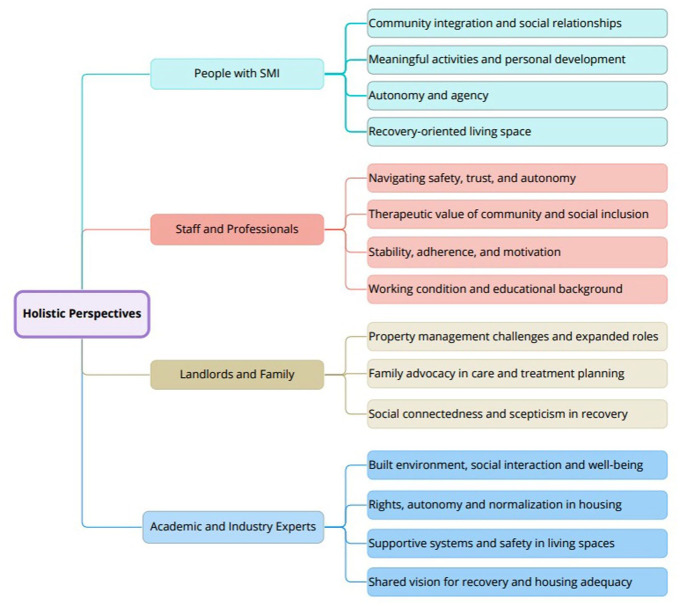
Overview of holistic perspective.

Swedish studies explored a broad range of housing types and innovative practices. Canadian and Dutch studies focused on recovery-oriented assisted independent housing. Australian and Swiss studies involved outreach and Housing First models. Perspectives of people with SMI featured in 81% of studies, mental health professionals in 46%, family or landlords in 12%, and academic or industry experts in 16%. Figure 2 shows thematic groupings by stakeholder.

### Perspectives of People With SMI (*n* = 21 studies)

#### Community Integration and Social Relationships

In two studies, people with SMI consistently valued community integration and social connection (Mötteli et al., 2022; Steno & Jønsson, 2023). Lindström et al. (2013) found that staff efforts to foster social participation in shared housing were notable, while Brunt and Rask (2018) identified trust in support staff as key to success (Brunt & Rask, 2018). Regular resident meetings were found to enhance social relationships and inclusivity (Rönngren et al., 2018; Steno & Jønsson, 2023). Meaningful support and community connection were linked to improved mental health (Fossey et al., 2020; Hill et al., 2010; Marcheschi et al., 2013). In independent housing, bi-weekly worker visits addressed social isolation (De Heer-Wunderink et al., 2012), while humane, supportive environments were more likely to foster a sense of home (Felx et al., 2020). van Genk et al. (2024) reinforced these findings, identifying trust, recognition, relationships, and belonging as central to recovery.

#### Meaningful Activities and Personal Development

Engagement in meaningful activities supported satisfaction, agency, and personal growth, contributing to a sense of home (Brunt & Rask, 2018; Eklund & Tjörnstrand, 2022; Forchuk et al., 2023). Felx et al. (2020) emphasised the importance of practical support to encourage such engagement. The ‘Active in My Home’ program, led by occupational therapists, improved resident satisfaction (Eklund & Tjörnstrand, 2022) and sense of purpose (Forchuk et al., 2023). Volunteering and hobbies enhanced motivation and self-worth (van Genk et al., 2024), while group activities promoted belonging, wellbeing, and goal achievement (Lindström et al., 2011; Lindström et al., 2013; Rönngren et al., 2018). Although Exergames increased activity, it had limited mental health benefits (Gyllensten & Forsberg, 2017). Diary analysis by Tjörnstrand et al. (2020) linked environmental improvements to higher housing satisfaction, though De Heer-Wunderink et al. (2012) found independent living sometimes correlated with dissatisfaction in daily life.

#### Autonomy and Agency

Independent living or cohabiting with chosen companions enhanced autonomy and a sense of belonging (Fossey et al., 2020; Marcheschi et al., 2014). Residents valued making their own daily decisions while receiving appropriate support (Forchuk et al., 2023; Lindström et al., 2011; Piat et al., 2020). In both Australia and Sweden, participants preferred self-contained units that offered privacy and allowed personalisation (Hill et al., 2010; Lindström et al., 2013). Privacy was linked to greater self-efficacy and housing satisfaction (Felx et al., 2020; Lindström et al., 2013). van Genk et al. (2024) highlighted the role of flexible, responsive supports in empowering individuals to make autonomous choices central to recovery

#### Recovery-oriented Living Space

Appealing design, maintenance, and access to green space were found to enhance self-perceptions of recovery (Marcheschi et al., 2013; Piat et al., 2017) extended the concept of home to surrounding therapeutic landscapes. Open, communal areas supported staff–resident interaction (Forchuk et al., 2023; Tjörnstrand et al., 2020), while design features such as privacy, tranquillity, and environmental satisfaction were linked to empowerment and participation (Felx et al., 2020; Fossey et al., 2020; Lindström et al., 2011). A homely atmosphere with sufficient space and privacy aided daily functioning and recovery (Eklund & Tjörnstrand, 2022; Forchuk et al., 2023). Safe-feeling housing was also key to symptom management for people with schizophrenia (Hill et al., 2010). van Genk et al. (2024) reinforced these insights, highlighting the value of peaceful, private, and stable living environments. Perceptions of housing quality was strongly associated with better recovery support (Eklund & Tjörnstrand, 2022; Marcheschi et al., 2014).

### Perspectives of Staff and Professionals (*n* = 12 studies)

#### Navigating Safety, Trust, and Autonomy in Therapeutic Relationships

Staff perspectives on safety, trust, and autonomy varied in relation to on-site experiences. Brunt and Rask (2018) identified a mismatch in priorities: on-site residential staff emphasised safety, while residents prioritised being able to trust the staff. Molin et al. (2023) found that visiting nurses perceived on-site staff as inflexible and resistant to collaboration. These dynamics negatively affected residents’ sense of home. Both studies emphasised that collaborative, cohesive staff relationships modelled inclusion and fostered a more therapeutic environment. Nonetheless, tension between safeguarding and fostering trust reflects broader power dynamics in professional care roles where the imperative to manage risk can at times conflict with promoting autonomy and relational openness.

#### Therapeutic Value of Community and Social Inclusion

Brunt and Rask (2018) found that people with SMI benefited when staff supported daily tasks and accompanied them into the wider community. Adamus et al. (2023) argued that support in the community to learn social cues and interaction skills in real-work settings enhanced individuals’ community experiences. Both studies highlighted that sense of home was closely tied to enjoyment and connection within the surrounding community. In both studies, home was considered reliant on pleasure in the surrounding community. However, social stigma remained an obstacle, negatively affecting self-perception and motivation to venture outside (Adamus et al., 2023).

#### Stability, Adherence, and Motivation

Adamus et al. (2023) reported that workers linked housing quality to behaviour, and behaviour to mental health stability. In Brunt and Rask (2018), staff gave residents direct feedback when their behaviours affected others. Both studies highlighted interactions between personal motivation, adherence to treatment, and mental health outcomes, suggesting that honest, supportive feedback enhanced social interactions and, in turn, improved both living conditions and housing experiences (Adamus et al., 2023; Brunt & Rask, 2018).

#### Working Condition and Educational Background of Professionals

Adamus et al. (2023) compared working conditions and educational backgrounds of staff in independent assisted housing and residential care. Residents in these settings experienced a stronger sense of home, improved wellbeing, and better relationships with staff than those in residential care. Adamus et al. (2023) concluded that professional value-driven, low-intensity support was essential for effective housing and rehabilitation outcomes.

### Perspectives of Landlords and Family Members (*n* = 3 studies)

#### Property Management Challenges and Expanded Roles

Landlords reported challenges renting to tenants with SMI due to conflict, property damage, hygiene concerns, and behavioural issues (Bengtsson-Tops et al., 2014). They often mediated conflicts with other tenants and neighbours, taking on roles beyond typical property management. Many expressed feeling unsupported by mental health services when managing these complex situations without guidance or resources. Bengtsson-Tops et al. (2014) advocated for systemic change, urging collaboration between landlords and mental health services to support tenants making a home in the private rental market. Overlap between tenancy management and psychosocial support shows how non-clinical actors can become drawn into care roles, raising questions about responsibility, power, and whether systems adequately support housing stability and tenant autonomy, both required to make a home.

#### Family Advocacy in Care and Treatment Planning

One study in Sweden reported frustration by family members in their difficulties accessing supports for loved ones with SMI, living in supported independent housing (Felx et al., 2020). Using multidimensional scaling and hierarchical cluster analysis, the study highlighted the crucial role of families in providing support. However, families sought greater involvement in care planning and recognition of their contribution to creating a home-like environment as part of recovery – advocating for their loved ones greater independence alongside wanting to retain influence over care decisions in service contexts where formal services were perceived as insufficient. Both family members and staff stressed the need for respite, quality time off, and self-care, given the high demands of caregiving. Such dynamics also reflect broader tensions in power-sharing between informal caregivers and professional systems.

#### Social Connectedness and Scepticism in Recovery

Family members in Piat and Seida (2018) emphasised the need for coordinated efforts between family and on-site staff to balance support with autonomy – an approach also reflected in the views of people with SMI (De Heer-Wunderink et al., 2012; Lindström et al., 2013; van Genk et al., 2023). In Piat and Seida (2018), family members wanted staff to assist their loved ones in forming meaningful social connections in the community to improve self-identity and self-worth. While generally supportive of autonomy, many family members in this study remained sceptical of ‘full’ recovery.

### Perspectives of Academic and Industry Experts (*n* = 4 studies)

#### Built Environment, Social Interaction, and Wellbeing

Marcheschi et al. (2014, 2016), through quantitative and qualitative studies, found that academics, user-group panel members, and others strongly believed the built environment shapes social interaction and wellbeing. Well-defined private and shared spaces, ample amenities, and quality outdoor areas were shown to support positive resident interactions. In contrast, poorer-quality housing, often centrally located and lacking green spaces, was linked to fewer positive exchanges. The authors concluded that thoughtfully designed environments, both inside and out, could enhance social interaction, overall wellbeing, and feeling ‘at home’.

#### Rights, Autonomy, and Normalisation in Housing

Academic and industry experts in van Genk et al. (2023) expressed the importance of normalisation housing, where people have autonomy, privacy, and opportunities for engaging with others in pleasing surroundings. The authors emphasised housing as a fundamental right, drawing from key themes of equitable opportunity and the right to define one’s own path in advocating for ‘normalisation’ in the context of ‘citizenship’ and ‘recovery’. Calling for rights-based living environments, van Genk et al. (2023) emphasised collaboration among family, peers, professionals, and flexibility in the provision of around-the-clock support. They introduced the concept of ‘equivalence’, stating that supports needed to respond uniquely to the aspirations of people with SMI, stressing the importance of active participation rather than being passive recipients of assistance.

#### Supportive Systems and Safety in Living Spaces

Academic and industry experts highlighted the importance of creating genuinely homelike living spaces. Participants in van Genk et al. (2023) and Felx et al. (2020) underscored the need for safety, security, and protection from stigma in the home. To achieve these ends, findings suggested that prioritising staff training on person-centred care was more effective than cost-driven mental health approaches. Participants in these studies agreed on the need for public education to reduce stigma and improved communications with landlords were critical components. Additionally, collaboration between families, mental health workers, and other services were perceived as vital for optimising living conditions in assisted independent housing.

#### Shared Vision for Recovery and Housing Adequacy

Studied in Canada, Felx et al. (2020) highlighted that managers and administrators perceived a shared vision among stakeholders as crucial for improving housing quality and living experiences. Key features for achieving this endeavour were how to resolve discrepancies in housing quality assessments made by managers, service users, families, and policymakers across different housing contexts and settings. Acknowledging the challenges of affordable housing, ‘managers and administrators’ noted that rental subsidies and assistance in finding suitable roommates could enhance living conditions. However, Felx et al. (2020) stressed, that a shared commitment from diverse stakeholders who were willing to support the housing needs of people with SMI was essential to ensuring they could live fulfilling lives in homes within their communities.

## Discussion

This review aligns with global efforts to optimise the sense of home for people with SMI. The findings challenge prevailing models that treat housing as merely a logistical or clinical issue, focusing on placement, risk management, or service provision. Instead, it underscores that home is not static or confined to physical shelter or tenancy (Doroud et al., 2018; Padgett, 2007). Our synthesis supports the view of home as dynamic, relational, and socially constructed, evolving through ongoing interactions between individuals and their environment. This perspective resonates with the socio-ecological model (Bronfenbrenner, 2000), which complicates individualised recovery models by emphasising the role of multiple, interacting systems, from proximal relationships with staff and co-residents to broader factors like housing policies, service funding, and social stigma (Tsemberis et al., 2004; Ware et al., 2007).

The concept of ‘Ontological Security’ (Dupuis & Thorns, 1998; Padgett, 2007) emerged in participants’ emphasis on stability, safety, and control. Home was often described as a space to restore identity and meaning (Mallett, 2004), particularly when individuals could personalise their environment and establish routines. Several studies demonstrated that home transcended mere housing, with emotional comfort, respect, and belonging being vital for recovery (Somerville, 1992). Consistent with Padgett’s (2007) view of home as a recovery space, participants valued flexible support, privacy, and opportunities to pursue personal goals. Home was also shaped by everyday practices and interactions (Despres, 1991), though institutional rules or lack of privacy could disrupt this. The process of becoming a home involved ongoing negotiation (Blunt, 2005; Dowling & Mee, 2007), often supported by staff continuity and meaningful engagement involved ongoing negotiation and relational connection. When individuals had agency over their living environment, housing was more likely to promote wellbeing (Doroud et al., 2018; van Genk et al., 2024). Housing also offered a platform for social identity and community re-engagement (Rowe & Davidson, 2016), though systemic barriers such as stigma or restrictive tenancy models often constrained this. These findings align with theories like place attachment, social capital, recovery theory, and ecological systems, which emphasise the impact of environment, social networks, and supportive settings on mental health recovery (Anthony, 1993; Bronfenbrenner, 2000; Putnam, 2001; Scannell & Gifford, 2010).

Since deinstitutionalisation, housing interventions have evolved from custodial to supportive, and ultimately to person-centred, autonomy-focused assisted independent housing (Dorvil et al., 2005; Farkas & Coe, 2019) as emphasised in most studies in this review. Supported housing combines treatment and care, encouraging residents to recover their citizenship, manage mental health, develop social and living skills, and improve functioning. It encourages individual choice and independence, separating housing from mandatory treatments or services (Nelson, 2010; Parkinson et al., 1999). This indicates a clear shift in contemporary literature from a paternalistic and directive model to ones that emphasise individual rights, and personalised support, facilitating the transition from institutions to houses, and from houses to homes (Friedrich et al., 1999; Nelson, 2010; Nelson & Caplan, 2017). The transition towards personalised, flexible, and community-integrated models of care reflects global trends in mental health service provision (Kinchin et al., 2019; Macpherson et al., 2012).

In bringing together all four stakeholder group perspectives from across the studies reviewed, Figure 2 offers a comparative view showing distinct priorities and challenges with transforming the accommodation of people with SMI into homes. While autonomy and community integration may be universally valued, results highlight different perspectives on priorities across the 26 studies. Workers and professionals respect autonomy, while families desire greater involvement, and landlords seek guidance. Key challenges include social stigma and property management burdens, underscoring the need for collaborative efforts in housing provision and the making of home. There is also a division between perceptions of the role of education on physical environments and between aesthetic and practical housing considerations.

The review elucidates the complexity of ‘home’ for individuals with SMI, influenced by diverse stakeholder perspectives. It underscores, for SMI individuals, that home is more than a physical space – it is intrinsically linked with community integration, autonomy, and supportive environments. This view is reflected within placemaking literature, emphasising the role of community engagement, quality spaces, and personal relationships in fostering a sense of belonging (Chisholm et al., 2024; O’Donnell et al., 2024; Schneider, 2022). In shaping this sense of home, the quality of professional support, individual choice, and inclusive social environment are crucial, as are interactions and support from staff. While few published studies present the involvement of landlords and family members in social conditions and community interactions, their involvement in either supporting or hindering a sense of home for individuals with SMI is acknowledged. Expert guidance is key to ensuring safe, secure, and recovery-conducive living spaces.

In their review, O’Malley and Croucher (2005) echoed the challenge of fostering independence within housing programs, aligning their findings with the importance of professional support and inclusive environments in defining home. This highlights the necessity of holistic, collaborative approaches in housing, reconciling recovery-focused practices with risk-averse regulations, and advocating for policies that facilitate the recovery of citizenship.

## Conclusion

This review synthesised stakeholder perspectives making a house into a home in the context of SMI, highlighting the significance of social relationships, community integration, meaningful activities, personal development, and autonomy. It stresses the importance of trust and rapport, achieving balance between safety and self-determination, and the therapeutic benefits of social inclusion in fostering a sense of home. The review contributes insights on the therapeutic potential of built environments, activity and personal growth opportunities, and collaborative care and support practices. Adopting multi-stakeholder perspectives helps to respond to the complexities of living with SMI, promoting recovery, autonomy, and community integration. This will require coordinated efforts to create supportive, inclusive, and empowering environments, recognising multiple dimensions in recovering citizenship. Stakeholders should consider targeted landlord training in mental health awareness, designing adaptable and inclusive housing spaces, and strengthening multidisciplinary collaboration between housing providers, clinical teams, and community services to sustain tenancy and promote recovery-oriented environments.

We acknowledge several study limitations. Substantial heterogeneity in aims, interventions, and outcomes across studies hindered the identification of strong or consistent themes. With our primary interest in actual interventions that support making housing into a home, mostly qualitative data (or discussion in quantitative studies) assisted in achieving some insights. A lack of specific research on directly engaging with people to make housing into a home is also noted. As well, studies from different countries use inconsistent terminologies affecting interpretation and global applicability. Limited reporting on participant age also restricts understanding of whether and how experiences of home differ across life stages; future research should examine these potential differences explicitly. Despite these anomalies, greater involvement of people with SMI in intervention design and research is needed to ensure genuine insights. Intervention detail is also needed to better inform community services.

## Supplemental Material

sj-docx-1-isp-10.1177_00207640251387785 – Supplemental material for Making a Home for Individuals With Serious Mental Illness: A Systematic Review Supplemental material, sj-docx-1-isp-10.1177_00207640251387785 for Making a Home for Individuals With Serious Mental Illness: A Systematic Review by Emi Patmisari, Yunong Huang, Ros Wong, Mark Orr, Sumathi Govindasamy, Emily Hielscher and Helen McLaren in International Journal of Social Psychiatry
