# Supplementary material for: Making a Home for Individuals With Serious Mental Illness: A Systematic Review
Source: Int J Soc Psychiatry. 2025 Nov 4;72(4):745–59. doi: 10.1177/00207640251387785 (PMC13263474; doi:10.1177/00207640251387785)
Supplement: sj-docx-1-isp-10.1177_00207640251387785 – Supplemental material for Making a Home for Individuals With Serious Mental Illness: A Systematic Review [file sj-docx-1-isp-10.1177_00207640251387785.docx]

**Search Strategy**

CINAHL

| **#** | **Query** | **Search Options** | **Results** |
| --- | --- | --- | --- |
| S1 | “severe mental illness” OR “serious mental illness” OR smi OR schizophreni* OR psychotic OR psychos?s OR ptsd OR “post-traumatic stress” OR mdd OR “major depressi*” OR bipolar OR schizoaffective OR “eating disorders” OR schizoid OR “personality disorder” OR ocd OR “obsessive compulsive” | **Expanders** - Apply equivalent subjects  **Search modes** - Boolean/Phrase | 146,661 |
| S2 | home OR hous* OR dwelling OR residen* OR apartment OR flat OR accommodation OR rent* | **Expanders** - Apply equivalent subjects  **Search modes** - Boolean/Phrase | 437,533 |
| S3 | control OR determination OR authority OR routine OR daily OR privacy OR secur* OR freedom OR identit* OR stability OR belong* OR safe OR independen* OR autonomy OR skill OR confidence | **Expanders** - Apply equivalent subjects  **Search modes** - Boolean/Phrase | 2,322,735 |
| S4 | support OR intervention OR assistance OR service OR program OR “assisted living” OR “supported housing” OR “supported accommodation” OR “independent living” OR “living arrangement” | **Expanders -** Apply equivalent subjects  **Search modes -** Boolean/Phrase | 1,874,003 |
| S5 | S1 AND S2 AND S3 AND S4 | **Limiters** - English Language; Age Groups: Adolescent: 13-18 years, Adult: 19-44 years, Middle Aged: 45-64 years, Aged: 65+ years, Aged, 80 and over  **Expanders** - Apply equivalent subjects  **Search modes** - Boolean/Phrase | 1,352 |

Scopus

823 documents

TITLE-ABS ( ( "severe mental illness"  OR  "serious mental illness"  OR  smi  OR  schizophreni*  OR  psychotic  OR  psychos?s  OR  ptsd  OR  "post-traumatic stress"  OR  mdd  OR  "major depressi*"  OR  bipolar  OR  schizoaffective  OR  "eating disorders"  OR  schizoid  OR  "personality disorder"  OR  ocd  OR  "obsessive compulsive" )  AND  ( home  OR  hous*  OR  dwelling  OR  residen*  OR  apartment  OR  flat  OR  accommodation  OR  rent* )  AND  ( control  OR  determination  OR  authority  OR  routine  OR  daily  OR  privacy  OR  secur*  OR  freedom  OR  identit*  OR  stability  OR  belong*  OR  safe  OR  independen*  OR  autonomy  OR  skill  OR  confidence )  AND  ( support  OR  intervention  OR  assistance  OR  service  OR  program  OR  "assisted living"  OR  "supported housing" OR "supported accommodation"  OR  "independent living"  OR  "living arrangement" )  AND  ( adolescen*  OR  youth  OR  "young people"  OR  teen  OR  adult ) )  AND  ( LIMIT-TO ( LANGUAGE ,  "English" ) )

Web of Science

705 document results

“severe mental illness” OR “serious mental illness” OR smi OR schizophreni* OR psychotic OR psychos?s OR ptsd OR “post-traumatic stress” OR mdd OR “major depressi*” OR bipolar OR schizoaffective OR “eating disorders” OR schizoid OR “personality disorder” OR ocd OR “obsessive compulsive” (Abstract) AND home OR hous* OR dwelling OR residen* OR apartment OR flat OR accommodation OR rent* (Abstract) AND control OR determination OR authority OR routine OR daily OR privacy OR secur* OR freedom OR identit* OR stability OR belong* OR safe OR independen* OR autonomy OR skill OR confidence (Abstract) AND support OR intervention OR assistance OR service OR program OR “assisted living” OR “supported housing” OR “supported accommodation” OR “independent living” OR “living arrangement” (Abstract) AND adolescen* OR youth OR "young people" OR teen* OR adult (Abstract) and English (Languages)

PubMed

264 results

(((("severe mental illness"[Title/Abstract] OR "serious mental illness"[Title/Abstract] OR smi[Title/Abstract] OR schizophreni*[Title/Abstract] OR psychotic[Title/Abstract] OR psychos?s[Title/Abstract] OR ptsd[Title/Abstract] OR "post-traumatic stress"[Title/Abstract] OR mdd[Title/Abstract] OR "major depressi*"[Title/Abstract] OR bipolar[Title/Abstract] OR schizoaffective[Title/Abstract] OR "eating disorders"[Title/Abstract] OR schizoid[Title/Abstract] OR "personality disorder"[Title/Abstract] OR ocd[Title/Abstract] OR "obsessive compulsive"[Title/Abstract]) AND (home[Title/Abstract] OR hous*[Title/Abstract] OR dwelling[Title/Abstract] OR residen*[Title/Abstract] OR apartment[Title/Abstract] OR flat[Title/Abstract] OR accommodation[Title/Abstract] OR rent*[Title/Abstract])) AND (control[Title/Abstract] OR determination[Title/Abstract] OR authority[Title/Abstract] OR routine[Title/Abstract] OR daily[Title/Abstract] OR privacy[Title/Abstract] OR secur*[Title/Abstract] OR freedom[Title/Abstract] OR identit*[Title/Abstract] OR stability[Title/Abstract] OR belong*[Title/Abstract] OR safe[Title/Abstract] OR independen*[Title/Abstract] OR autonomy[Title/Abstract] OR skill[Title/Abstract] OR confidence[Title/Abstract])) AND (support[Title/Abstract] OR intervention[Title/Abstract] OR assistance[Title/Abstract] OR service[Title/Abstract] OR program[Title/Abstract] OR "assisted living"[Title/Abstract] OR "supported housing" [Title/Abstract] OR "supported accommodation"[Title/Abstract] OR "independent living"[Title/Abstract] OR "living arrangement"[Title/Abstract])) AND (adolescen*[Title/Abstract] OR youth[Title/Abstract] OR "young people"[Title/Abstract] OR teen*[Title/Abstract] OR adult[Title/Abstract])

PsycINFO

| **#** | **Searches** | **Results** |
| --- | --- | --- |
| S1 | ("severe mental illness" or "serious mental illness" or smi or schizophrenia or schizophrenic or psychotic or psychosis or ptsd or post-traumatic or mdd or "major depression" or "major depressive" or bipolar or schizoaffective or "eating disorders" or schizoid or "personality disorder" or ocd or "obsessive compulsive").mp. [mp=title, abstract, heading word, table of contents, key concepts, original title, tests & measures, mesh word] | 507,333 |
| S2 | (home or hous* or dwelling or residen* or apartment or flat or accommodation or rent*).m_titl. | 65,072 |
| S3 | (control or determination or authority or routine or daily or privacy or secur* or freedom or identit* or stability or belong* or safe or independen* or autonomy or skill or confidence).mp. [mp=title, abstract, heading word, table of contents, key concepts, original title, tests & measures, mesh word] | 1,412,821 |
| S4 | (support or intervention or assistance or service or program or "assisted living" or "supported housing" or "supported accommodation" or "independent living" or "living arrangement").mp. [mp=title, abstract, heading word, table of contents, key concepts, original title, tests & measures, mesh word] | 1,185,042 |
| S5 | S1 AND S2 AND S3 AND S4 | 932 |
| S6 | limit 5 to (english language and (adolescence <13 to 17 years> or adulthood <18+ years>)) | 785 |

ProQuest

953 results

abstract(“severe mental illness” OR “serious mental illness” OR smi OR schizophreni* OR psychotic OR psychos?s OR ptsd OR “post-traumatic stress” OR mdd OR “major depressi*” OR bipolar OR schizoaffective OR “eating disorders” OR schizoid OR “personality disorder” OR ocd OR “obsessive compulsive” ) AND abstract(home OR hous* OR dwelling OR residen* OR apartment OR flat OR accommodation OR rent* ) AND abstract(control OR determination OR authority OR routine OR daily OR privacy OR secur* OR freedom OR identit* OR stability OR belong* OR safe OR independen* OR autonomy OR skill OR confidence) AND abstract(support OR intervention OR assistance OR service OR program OR “assisted living” OR “supported housing” OR “supported accommodation” OR “independent living” OR “living arrangement” ) AND abstract(adolescen* OR youth OR "young people" OR teen* OR adult ) Applied filters: English
